# Tailored Direct Oral Anticoagulation in Patients with Atrial Fibrillation: The Future of Oral Anticoagulation?

**DOI:** 10.3390/jcm11216369

**Published:** 2022-10-28

**Authors:** Matej Samoš, Tomáš Bolek, Lucia Stančiaková, Martin Jozef Péč, Kristína Brisudová, Ingrid Škorňová, Ján Staško, Marián Mokáň, Peter Kubisz

**Affiliations:** 1 Department of Internal Medicine I, Jessenius Faculty of Medicine in Martin, Comenius University in Bratislava, 03659 Martin, Slovakia; 2 National Centre of Hemostasis and Thrombosis, Department of Hematology and Blood, Transfusion, Jessenius Faculty of Medicine in Martin, Comenius University in Bratislava, 03601 Martin, Slovakia

**Keywords:** direct oral anticoagulants, tailored medicine, DOAC laboratory monitoring, atrial fibrillation, adverse thrombotic and hemorrhagic events

## Abstract

Direct oral anticoagulants (DOAC) are currently the drug of choice for drug prevention of stroke or systemic embolism in patients with atrial fibrillation (AF). However, repeated ischemic stroke or systemic embolism and bleeding while on DOAC is still a challenging clinical phenomenon in the management of future long-term anticoagulation. It is not known whether tailoring the DOAC therapy to achieve optimal therapeutic drug levels could improve the clinical course of DOAC therapy. To be able to tailor the therapy, it is necessary to have a valid laboratory method for DOAC level assessment, to be aware of factors influencing DOAC levels and to have clinical options to tailor the treatment. Furthermore, the data regarding clinical efficacy/safety of tailored DOAC regimes are still lacking. This article reviews the current data on tailored direct oral anticoagulation in patients with AF.

## 1. Introduction

Direct oral anticoagulants (DOAC) (Table 1), drugs directly inhibiting thrombin (dabigatran) or activated coagulation factor X (apixaban, edoxaban and rivaroxaban), are currently the drugs of choice for the pharmacological prevention of stroke or systemic embolism [1] in patients with atrial fibrillation (AF). Thrombosis (ischemic stroke or systemic embolism) and bleeding while on DOAC is still a challenging clinical phenomenon in the management of future long-term anticoagulation. Patients with ischemic stroke on DOAC have a high 90-day mortality (35.1% reported in previous study), with the majority of deaths due to the stroke itself [2]. The unfavorable clinical course can also be seen in DOAC-treated patients who suffer from adverse bleeding. For example, adjusted one-year mortality is significantly higher in patients who suffered from gastric bleeding on DOAC therapy compared with those who did not [3]. In addition, previous studies have demonstrated that these adverse events correlate with in-optimal plasma levels of DOAC [4,5,6]. The question is whether tailoring the DOAC therapy to achieve optimal therapeutic drug levels could improve the clinical course of DOAC therapy. The aim of this article is to review the current data about tailored direct oral anticoagulation in patients with AF.

## 2. In-Optimal DOAC Levels and the Risk of Future Adverse Events

As mentioned, currently there are quite convincing data regarding the association between the risk of future adverse events and in-optimal (too low or too high) plasma levels of DOAC in AF patients on long-term DOAC therapy. First, a sub-analysis of the RE–LY trial showed that, in this trial, the occurrence of stroke and adverse bleeding correlated with dabigatran plasma levels. In this trial, on average, individuals who had a major hemorrhagic event had higher trough and post-dose dabigatran levels than individuals who did not experience a bleeding event. In the multivariate analysis of ischemic stroke/systemic embolism, there was an inverse relation between dabigatran trough levels and the probability of an event. Second, Testa et al. reported in their studies [5,6] that bleeding during DOAC therapy was more frequent in AF patients with high peak drug levels [5] and thrombotic events developed in individuals who had low baseline trough drug levels [6]. Third, looking at the drug levels at the time of the bleeding or ischemic event [7,8], patients with a DOAC therapy-related bleeding had significantly higher and patients with a stroke despite taking DOAC had significantly lower DOAC levels at the time of this event compared to individuals tolerating the DOAC therapy without any adverse events. Finally, in a recent observational study performed by Siedler et al. [9] patients who suffered from early ischemic stroke recurrence despite the use of DOAC had low DOAC plasma levels (this was demonstrated for apixaban and dabigatran after propensity score matching). In summary, the current evidence suggests an association between DOAC plasma levels and the risk of future adverse events, and that monitoring the DOAC levels may help to identify patients with increased risk for these events. The following questions should be answered: what method should be used for DOAC laboratory assessment and what are the optimal therapeutic levels for effective and safe DOAC therapy?

## 3. How to Measure DOAC Levels in AF Patients on Long-Term DOAC Therapy?

Although liquid chromatography-mass spectrometry (LC-MS) is still honored as a standard laboratory method for DOAC levels quantification [10,11,12], especially in the settings of preclinical/clinical research, there is a general consensus that the method is not very useful for the assessment of DOAC levels in routine clinical practice [13], mostly due to its limitations, such as bad availability, the need for specially equipped laboratory with specially skilled staff and time demands. Furthermore, standard coagulation test (prothrombin time, activated partial thromboplastin time, thrombin time) do not have sufficient sensitivity for DOAC levels assessment, especially when low DOAC levels are expected [10,13], and this could probably also be applied to standard reagents of novel viscoelastic hemostatic assays [14]. Therefore, DOAC-specific coagulation assays (ecarin clotting time assay or diluted thrombin time assays for dabigatran, and drug-specific chromogenic anti-Xa assays for apixaban, edoxaban and rivaroxaban) are arguably the most appropriate tests from the currently available laboratory methods for routine DOAC levels assessment (Table 2), as the assays demonstrated good correlation with LC-MS [11,12] and good clinical utility in previous post-marketing studies [15,16,17]. Nevertheless, the assays could be inaccurate at very low DOAC levels [10], and clinicians should always be aware of the limitations of DOAC laboratory testing when interpreting the results and choosing future strategies [13]. Although preliminary experience with novel thrombin generation assays [18] or novel automated thromboelastography [19] are promising, there is currently no sufficient evidence to recommend the use of these assays to guide clinical decisions in DOAC-treated patients [13].

The next, clinically important but yet not fully answered point is the question of the optimal timing of blood sampling for DOAC levels testing. As the majority of the published studies to date have reported an association between trough (pre-drug dose) and/or peak (post-drug-dose) drug levels with the risk of adverse events, it is quite reasonable to assess both drug levels. Trough drug level should definitely be measured in individuals with severe renal function impairment, with extremely high body weight (body mass index > 40 kg/m^2^), in those with advanced age (elderly ones), and if a new DOAC-levels-modifying drug interaction is expected [13]. As all commercially available DOAC reach their drug steady-state within the first two days after starting therapy, it is probably reasonable to test DOAC levels on the forth to fifth day after the drug initiation (after five or more intakes), and to repeat the measurement whenever needed (new adverse bleeding or thrombosis, new decrease in renal/hepatic function, new possible drug interaction, questionable patient drug compliance, etc.). However, this recommendation is only based on the data from pharmacokinetic studies and on expert opinion [13], and more research in this area is still warranted.

## 4. Optimal DOAC Plasma Levels for Long-Term Anticoagulation

Another issue which should be resloved prior to recommending a tailored DOAC strategy is the issue of optimal therapeutic DOAC plasma levels for long-term anticoagulation. Therapeutic drug levels can probably be established, in part, for dabigatran. In the aforementioned sub-analysis of the RE–LY trial [4] which showed a correlation between dabigatran plasma levels and the risk of adverse events, patients with trough dabigatran levels > 210 ng/mL had a two-fold higher risk of dabigatran-related bleeding. Thus, dabigatran plasma level 210 ng/mL can likely be used as the upper limit for safe anticoagulation. Going further, patients with dabigatran trough levels < 28 ng/mL had a two-fold higher risk of adverse thrombosis; therefore, dabigatran plasma levels of at least 28 ng/mL appear to be necessary for efficient anticoagulation. Nevertheless, these levels were established based on the results of a sub-analysis of a single phase III clinical trial, as no other study dealing with this issue is currently available. Testa et al. were not able to establish cut-off limits for the risk of adverse ischemic [6] or bleeding [5] events due to the low patient sample and low rate of adverse events. In our previous studies, which aimed to establish DOAC plasma levels at the time of an adverse event, in dabigatran-treated patients with bleeding, dabigatran levels of 261.4 ± 163.7 ng/mL were determined on average [7]. In patients with embolic stroke, average dabigatran plasma levels of 40.7 ± 36.9 ng/mL were found [8]. This observation probably supports the upper reference range of 210 ng/mL in terms of safety, but questions the lower reference range of 28 ng/mL in terms of efficacy.

This issue remains unexplained for apixaban, edoxaban and rivaroxaban at present. Sakaguchi et al. [20] showed, in their analysis of Japanese rivaroxaban-treated patients with bleeding complications, higher peak rivaroxaban levels (anti-Xa activity), and peak rivaroxaban levels independently predicted bleeding. In another interesting retrospective study, rivaroxaban trough deficiency (defined as trough rivaroxaban levels < 12 ng/mL) was associated with an increased risk of thrombotic events (but not bleeding) in Chinese patients with AF [21]. Additionally, Sin et al. [17] showed, in their prospective study enrolling rivaroxaban-treated AF patients with different stages of chronic kidney disease (Stage 1–3), that rivaroxaban trough levels in those with hemorrhage were higher (59.9 ± 35.6 ng/mL) than in those who were free from a bleeding episode (41.1 ± 29.2 ng/mL; *p* < 0.05). This study enrolled only 92 patients. In our previous analyses [7,8], there were significantly higher rivaroxaban levels at the time of bleeding compared to the trough levels of patients who did not have complications during rivaroxaban administration (245.9 ± 150.2 ng/mL versus 52.5 ± 36.4 ng/mL; *p* < 0.001), and rivaroxaban levels tended to be lower in those experiencing embolic stroke (42.7 ± 31.9 ng/mL); however, the variability in drug plasma levels was the highest in rivaroxaban-treated patients. For apixaban, Limcharoen et al. [22] reported an association between apixaban trough levels and the risk of bleeding. In apixaban-treated patients with bleeding, apixaban trough levels of 139.15 ng/mL were reported. These levels are lower than our previous observation of apixaban levels of 311.8 ± 142.5 ng/mL at the time of a bleeding event [7]. It is interesting that, in the study performed by Limcharoen et al., almost all the patients presented apixaban plasma levels within the expected range, which was defined as a range of 34.0–230.0 ng/mL for trough and 69.0–321.0 ng/mL for peak drug levels [22]. The ranges were derived from a pharmacokinetic study with apixaban [23]. Unfortunately, there is no other study dedicated to the relationship between apixaban plasma levels and the risk of adverse ischemic or bleeding events (except for the previously mentioned ones performed by Testa et al. [5,6]), and no such study for edoxaban.

Summarizing this issue, the determination of optimal DOAC plasma levels for long-term anticoagulation still needs further research (especially for rivaroxaban, apixaban and edoxaban); nevertheless, levels derived from pharmacokinetic studies should probably not be used, as these data report only the expected drug level when a defined dose of the drug is taken, but do not correlate with the risk of bleeding or thrombosis during long-term therapy.

## 5. Factors Influencing DOAC Plasma Levels

**Figure 1:**
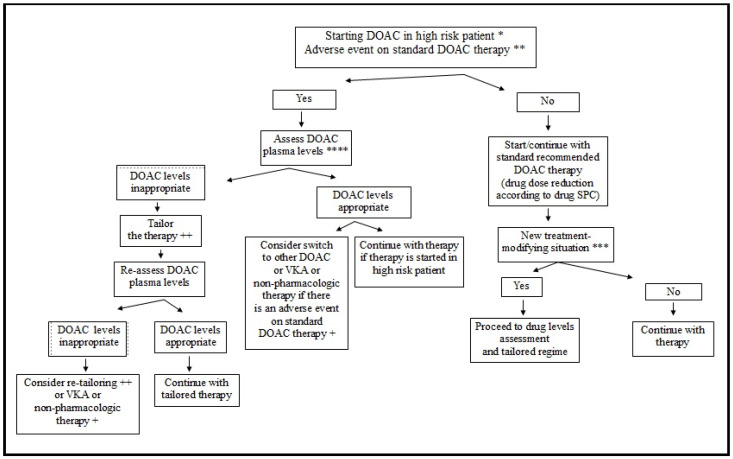
A proposed scheme for tailored direct oral anticoagulation. DOAC—direct oral anticoagulants; INR—international normalized ratio; LC-MS—liquid chromatography-mass spectrometry; SPC—summary of product characteristics; VKA—vitamin K antagonists. * severe renal/hepatic function impairment, extremely high body weight, advanced age, drug interactions. ** embolic stroke/systemic embolism or bleeding on labeled DOAC therapy. *** new adverse bleeding or thrombosis, new decrease in renal/hepatic function, new possible drug interaction, questionable patient drug compliance. **** LC-MS (if available) or drug-specific coagulation assays; test for trough (pre-drug dose) and peak (post-drug dose) levels. ^+^ VKA with target INR 2-3 or left atrial appendage (surgical/transcatheter) occlusion. ^++^ by optimizing drug dose or switch strategy or by modification of (modifiable) factors influencing DOAC drug levels.

When deciding on a tailored DOAC strategy (Figure 1), one should be aware of clinical features/factors that could possibly influence DOAC plasma levels (Table 1). Looking at the currently available data [24], DOAC levels could be changed (increased) in patients with a reduced glomerular filtration rate (to a lesser extent in case of apixaban administration), in elderly individuals (increased, especially when dabigatran is used) [25,26,27], and there are several relevant drug interactions [28] leading either to a change in gastric pH, which is important for the absorption of dabigatran [29], or to changed P-glycoprotein (P-gp) or cytochrome P450 (CYP) activity, which could, in theory, affect the pharmacokinetics of all the available DOAC (P-gp) or the pharmacokinetics of oral factor Xa inhibitors (CYP) [28]. At present, it is not entirely clear whether extreme body weight (extremely high or extremely low) affects DOAC plasma levels. In their retrospective analysis, Piran et al. [30] reported that most of the patients with body weight over 120 kg had peak plasma levels higher than the median trough level for each of the three DOAC (apixaban, dabigatran, rivaroxaban); but 21% of patients had a peak plasma concentration that was below the usual on-therapy range. On the other side, the authors of another prospective study showed that patients with extreme obesity (mean body mass index 44.4 kg/m^2^) and AF who were receiving DOAC therapy had DOAC plasma levels, within the expected range [31]. Obesity did not affect the plasma levels of apixaban and rivaroxaban in another prospective study (in patients with venous thromboembolism) [32], and plasma levels of apixaban in a previously published case of morbidly obese patient treated for AF [33]. Data from post-marketing studies regarding the DOAC levels in patients with extremely low body weight are still lacking. In addition, several studies suggested a possible role of genetic polymorphism in several candidate genes (CES1 gene encoding plasmatic esterase for dabigatran; ABCB1 gene encoding P-gp for apixaban, dabigatran, rivaroxaban and edoxaban; and SLCO1B1 gene encoding organic anion transporter protein 1B1 for edoxaban); however, the results of the studies published to date are controversial [34,35,36,37,38].

## 6. How to Tailor DOAC Therapy

Another issue is the question of optimal approach for tailoring the DOAC therapy. In theory, DOAC therapy might be tailored by optimizing the drug dose (increasing/decreasing) or by switching the drug. None of these approaches have been validated in clinical trials. The strategy of tailoring the drug dose has a possible disadvantage in the use of a drug dose that is higher than the dose tested in clinical trials (for example if there is a need to increase the dose in a patient already taking dabigatran 150 mg twice daily or apixaban 5 mg twice daily). Similarly, if there is a need to reduce the dose in a patient already taking a reduced drug dose (for example, the need to reduce drug dose in a patient taking 15 mg of rivaroxaban or 30 mg of edoxaban daily), the second reduction will lead to a drug dosing thatwas not previously tested in the settings of prevention of stroke or systemic embolism related to AF. Therefore, the second strategy (switch strategy) seems to be more favorable, as it does not have the disadvantage of off-label drug dosing. On the other hand, in a recent study performed by Suwa et al. [39], a laboratory monitoring based an off-label underdosing of rivaroxaban and apixaban in selected patients did not lead to an increased risk of bleeding or thromboembolic events during follow up, and achieved acceptable peak drug levels (155–400 ng/mL for rivaroxaban, 90–386.4 ng/mL for apixaban, respectively). Nevertheless, only 73 patients used off-label underdosed rivaroxaban and only 46 patients used off-label underdosed apixaban, and there is a strong risk of selection bias. All these disadvantages should be taken into account when interpreting the results of this study. Another possible way of tailoring DOAC therapy is to try to modify the modifiable factors influencing DOAC drug levels. For example, DOAC levels could be optimized by the reduction of possible food and drug interactions. In addition, non-pharmacologic procedures, such as left atrial appendage occlusion, could be further use to reduce the risk of stroke if pharmacological prophylaxis is difficult to manage [1].

## 7. Is It Possible to Improve DOAC Therapy by a Tailored Strategy?

To answer this question, it is important to validate the hypothesis that tailoring the DOAC therapy according to DOAC plasma levels detected by a laboratory monitoring would lead to reduced incidence of future thromboembolic and bleeding events in a randomized clinical trial. That trial should randomize AF patients with the need for long-term anticoagulation, fulfilling criteria for DOAC administration to either standard treatment regimen (fixed dosing according to drug summary of product characteristics, without any laboratory assessment of drug levels) or to a tailored treatment regimen (with laboratory assessment of DOAC dosing and either modifying the drug dose or switching to other DOAC if in-optimal DOAC levels are detected). The primary outcome of the trial should be the incidence of stroke or systemic embolism and bleeding during the follow- up period. To date, no study with this design is on-going or planned. Therefore, there is no direct evidence that a tailored DOAC strategy would lead to improved clinical outcomes of long-term DOAC therapy, and the question: “Is it possible to improve DOAC therapy by a tailored strategy?” remains unanswered. However, as several factors that may significantly affect DOAC plasma levels have been identified, the tailored regime can be considered in selected risk patients, such as those experiencing stroke despite labeled DOAC anticoagulation, those with repeated bleeding while on DOAC therapy, those with a need for combined antiplatelet and anticoagulant therapy and those with multiple risk factors for in-optimal DOAC drug levels [24,40]. However, the risk/benefit ratio should be carefully evaluated before making the decision to use a tailored DOAC strategy.

## 8. Conclusions

Considering the aforementioned data, unanswered issues and possible limitations, at present, tailored DOAC therapy should not be recommended as a routine strategy in clinical practice. The tailored regime should be used with caution, only in selected patients, and after an appropriate evaluation of the risk/benefit ratio. However, further research of tailored DOAC strategy should definitely be advocated.

## Figures and Tables

**Table 1:** Direct oral anticoagulants: targets, indications, and pharmacology.

Parameter	Apixaban	Dabigatran	Edoxaban	Rivaroxaban
Target	Factor Xa	Thrombin (Factor IIa)	Factor Xa	Factor Xa
FDA-approved indications	Nonvalvular AF, VTE (treatment *, secondary prevention, prophylaxis ^‡^)	Nonvalvular AF, VTE (treatment ^||^, secondary prevention, prophylaxis)	Nonvalvular AF, VTE (treatment ^§^)	Nonvalvular AF, VTE (treatment *, secondary prevention, prophylaxis ^‡^)
Safety in nonvalvular AF	Lower risk of major bleeding than with warfarin	Higher risk of GI bleeding than with warfarin	Lower risk of major bleeding than with warfarin; higher risk of GI bleeding (60 mg dose) than with warfarin	Higher risk of GI bleeding than with warfarin
Specific reversal agent	Andexanet alpha (specific for all factor Xa inhibitors)	Idarucizumab	Andexanet alpha (specific for all factor Xa inhibitors)	Andexanet alpha (specific for all factor Xa inhibitors)
Half-life (hours)	12	8–15	10–14	7–11
Renal clearance (%)	25	80	50	33
Dialyzable	No	Yes	No	No
Prodrug	No	Yes	No	No
Bioavailability (%)	60	6	62	60–80
Time to peak effect (hours)	1–2	1–3	1–2	2–4
Gene polymorphism studied	ABCB1	CES1, ABCB1	ABCB1, SLCO1B1	ABCB1
Non-pharmacologic interactions	Age, reduced body weight, reduced GFR (only if two conditions are simultaneously present), probably severe liver damage	Age, reduced GFR (do not use if eGFR < 30 mL/min/1.73 m^2^)	Reduced GFR (do not use if eGFR < 15 mL/min/1.73 m^2^), probably severe liver damage	Age (although dose reduction is not recommended), reduced GFR (do not use if eGFR < 15 mL/min/1.73 m^2^), probably severe liver damage
Drug interactions	Avoid apixaban with concomitant use of dual P-gp and moderate CYP 3A4 inhibitors	Dose reduces dabigatran with concomitant P-gp inhibitor, be cautious when gastric acidity reducing drugs are administered	Avoid concomitant use of rifampin; No adjustments for concomitant P-gp inhibitors	Avoid rivaroxaban with concomitant use of dual P-gp and moderate CYP 3A4 inhibitors

AF—atrial fibrillation; CYP—cytochrome P450; eGFR/GFR—(estimated) glomerular filtration rate, FDA—food and drugs administration; GI—gastrointestinal; P-gp—glycoprotein P; VTE—venous thromboembolism. * Twice daily for the first 21 days of VTE treatment; once daily for other indications for rivaroxaban or twice daily for apixaban. ^‡^ Approved for VTE prophylaxis after knee or hip surgery only. ^§^ Prophylaxis of VTE in adult patients hospitalized for an acute medical illness and for extended use. ^||^ After 5–10 days of parental anticoagulant treatment only.

**Table 2:** Specific assays for determination of DOAC levels/activity.

Test	Dabigatran	Rivaroxaban	Apixaban	Edoxaban	Note
Liquid chromatography-mass spectrometry (LC-MS)	↑↑↑	↑↑↑	↑↑↑	↑↑↑	Standard method for preclinical/clinical research, the most accurate method (especially when low levels are expected); limited usefulness in clinical practice
Diluted thrombin time assay	↑↑	x	x	x	Good correlation with LC-MS, lower accuracy if drug levels are low; good usefulness in clinical research/practice
Ecarin clotting time assay	↑↑	x	x	x	Good correlation with LC-MS, lower accuracy if drug levels are low; good usefulness in clinical research/practice
Drug-specific chromogenic anti-Xa assays	x	↑↑	↑↑	↑↑	Good correlation with LC-MS, lower accuracy if drug levels are low; good usefulness in clinical research/practice
Thrombin generation assays	↑↑	↑↑	↑↑	↑↑	Promising method, but not validated; usefulness in clinical research
Automated thromboelastography with drug-specific reagents	↑↑	↑↑	↑↑	not known/ not examined	Promising method, but not validated; usefulness in clinical research

↑—sensitivity (↑↑—good, ↑↑↑—excellent), x—not sensitive.

## Data Availability

All the source data are available at the Corresponding Author uppon a request.
